# Safety and Feasibility of Endovascular Management of Pediatric Dialysis Access

**DOI:** 10.1007/s00270-025-04070-2

**Published:** 2025-06-09

**Authors:** Jake DiFatta, Chase Mahler, Junjian Huang, A. J. Gunn, Junaid Raja

**Affiliations:** 1 School of Medicine, University of Alabama at Birmingham, Birmingham, AL USA; 2 Division of Vascular and Interventional Radiology/Department of Radiology, University of Alabama at Birmingham, 625 19Th St S, Suite 484, Birmingham, AL 35233 USA

**Keywords:** Pediatric dialysis, Angioplasty, Fistula, Graft patency

## Abstract

**Purpose:**

To investigate the safety and efficacy of pharmacomechanical intervention in the maintenance of pediatric dialysis access fistulas and grafts in the extremities.

**Materials and Methods:**

A retrospective analysis of 67 interventions performed on 17 pediatric patients with dialysis access maintenance interventions was conducted. Use of angioplasty, stenting, thrombectomy devices, and thrombolytic agents such as tissue plasminogen activator (tPA) were recorded across interventions. Total number of interventions per patient and time between reinterventions were measured. Safety of these techniques was assessed according to the Cardiovascular and Interventional Radiology Society of Europe (CIRSE) complication guidelines. Technical success was defined as restoration of patency without a hemodynamically significant stenosis, and clinical success was defined as symptom resolution without reintervention of greater than 6 months.

**Results:**

The application of pharmacomechanical intervention demonstrated a favorable safety profile and high technical success rates across all categories. The median age at first intervention was 13 years (IQR 10.1–16.9, range 7–18), and median number of interventions was 2 with a range of 1–11. No major complications were observed during or after the procedures. Among the 67 interventions, angioplasty was the most frequently employed technique (*n = *67, 100%), followed by thrombolysis and/or anticoagulation (*n = *34, 50.7%), thrombectomy (*n = *21, 31.3%), and stenting (*n = *10, 14.9%). Technical success was 98.5% (66/67), and combined 6-month primary assisted and secondary patency from the index dialysis circuit intervention was 41.8% (28/67). For patients who received more than one treatment, median time to reintervention was 107 days.

**Conclusion:**

The high rate of technical success and absence of major or minor complications suggest that endovascular techniques for dialysis access maintenance including angioplasty, stenting, thrombolysis, and advances therapies can be safely and effectively performed in pediatric patients. Rates of primary and secondary patency are slightly lower compared to the adult population.

**Level of Evidence:**

Level 4, Retrospective Cohort Study.

**Graphic Abstract:**

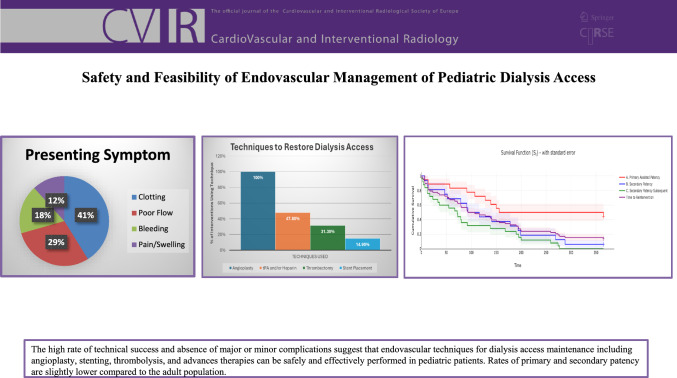

## Introduction

There are three principal treatment options for patients with end-stage renal disease (ESRD): transplant, hemodialysis, and peritoneal dialysis. In the adult population, roughly 28% of patients undergo transplantation, 63% are treated via hemodialysis and 9% via peritoneal dialysis [1]. However, treatment looks much different for children in the USA: 71% of children undergo transplantation, while 17% are treated via hemodialysis and 11% via peritoneal dialysis [2]. Therapies and protocols for maintenance of dialysis accesses are better described in the adult population, but scant literature exists concerning pediatric patients. The prevalence of pediatric end-stage renal disease is very low, with only 10–15 cases per one million children (0.001%), while roughly 2 out of 1000 (0.2%) US adults have ESRD a difference of 200-fold [3, 4]. Although the goal is almost always transplantation in pediatric patients, there are often barriers and delays encountered, necessitating dialysis as a bridge in such cases [5]. Transplantation offers improved quality of life and long-term survival, though for reasons including organ availability and patient compatibility, dialysis maintains a critical life-sustaining role during waiting periods [6, 7].

The disparate etiology of pediatric versus adult chronic kidney disease (CKD) impedes translation of treatment guidelines and standardized protocols from the adult to pediatric CKD population [8]. Diabetes and hypertension account for over half of the prevalence of CKD in adults [4]. In contrast, congenital and inherited disorders are responsible for about two thirds of all pediatric cases of CKD, with the most common cause being congenital abnormalities of the kidneys and urinary tract (CAKUT) [2, 8]. Children are thought to have higher rates of local intimal hyperplasia following intervention, which impact the durable clinical success of angioplasty and stent placement [9]. Pediatric clotting physiology is also distinct from that of adults; children produce less thrombin than adults and have roughly double the level of alpha-2-macroglobulin, an inhibitor of thrombin. Protein C production is also decreased in the early years of childhood [10]. Thus, medications and laboratory values must be age-modified. Post-treatment protocols for anticoagulation and antiplatelet regimens are discussed in the methods section of the study.

In adults undergoing hemodialysis, native fistulas have been shown to have significantly longer patency rates and lower rates of complication than synthetic grafts and are therefore preferred [1, 11]. Repeat intervention is often required to maintain or restore patency in both grafts and fistulas; graft patency has been reported to be 54% at one year, while patency of native fistulas was 75–91% at one year in the adult population [12]. Vascular dialysis access points are prone to complications such as stenosis and thrombus, most commonly within or near the anastomoses and inflow/outflow tracts [13, 14]. Vascular interventionalists can employ techniques including angioplasty, stent placement, thrombectomy, thrombolysis, fistulography, and embolization, under image guidance, in the maintenance and salvage of dialysis access points [11, 13].

This paper aims to evaluate the safety and efficacy of the endovascular therapies to maintain pediatric dialysis access.

## Methods

A retrospective cohort study assessing pediatric patients undergoing dialysis access maintenance interventions at a large tertiary care academic children’s hospital was performed with approval from the local institutional review board (IRB) as an exempt study. Demographic information and baseline radiographic attributes were extracted from the medical record from 7/1/2013 to 11/10/2023.

Pediatric patients carrying a diagnosis of CKD/ESRD requiring dialysis and a history of endovascular intervention of their dialysis access were included. Patients were excluded if they were over the age of 19 at initial treatment. Patients were classified by treatment type, access type, intraoperative findings, presenting symptoms, and clinical symptom improvement. Each procedure was categorized by whether angioplasty, stenting, heparin or tissue plasminogen activator (tPA) administration, and/or thrombectomy were employed. Procedure-specific details such as presenting symptoms were extracted from each patient’s first intervention. The number of interventions and time between interventions were important metrics. There was no formal clinical surveillance protocol; if patients remained asymptomatic with fully functioning dialysis post-intervention, no clinic visits were needed. Complications were categorized per adverse event classification guidelines from the Cardiovascular and Interventional Radiology Society of Europe (CIRSE) [15].

Technical success was defined as restoring functional patency of the fistula or graft with no residual findings of hemodynamically significant stenosis on post-intervention imaging. Primary assisted patency—defined as the interval from an intervention on a non-thrombosed dialysis circuit to a subsequent intervention—and secondary patency—defined as the interval from de-clotting of a thrombosed access to subsequent intervention—were assessed both independently and as a composite metric at the 6-month mark following the index intervention. The composite metric was used because the initial creation date of the dialysis circuit was not identifiable for all included patients. Clinical success was measured as the percentage of patients not requiring reintervention (primary assisted and secondary patency) within 6 months from prior intervention. For patients with symptom recurrence within the 6-month follow-up period, median time to reintervention was assessed, alongside which techniques were used in each patient’s first and subsequent interventions.

The technical approach for these patients included direct dialysis circuit access using a micro-puncture set with or without ultrasound guidance. Seldinger technique was then used for serial exchanges to position at least one antegrade (in the direction of blood flow through the circuit) sheath. Depending on the nature of the intervention as a fistulogram or a de-clotting procedure, either angiography was performed or a 0.035-inch wire with a 4-5-F catheter was advanced centrally beyond the thrombosed segment, respectively. For fistulograms, imaging was obtained with angiography antegrade and with balloon or manual compression retrograde to determine patency versus flow-limiting stenoses throughout the extent of the circuit. Transluminal balloon angioplasty was the first-line intervention in cases of flow-limiting stenosis. Stent placement served as a secondary technique to angioplasty and was indicated for treatment of symptomatic pain and/or swelling due to persistent stenosis refractory to angioplasty typically after at least one trial of angioplasty alone. For de-clots, techniques were operator dependent.

Thrombectomy was indicated for treatment of acute thrombosis within the fistula or graft. Clinical indicators of access-threatening thrombosis included abrupt cessation of bruit or thrill over the access site, swelling of the extremity, or difficulty cannulating the fistula, or indicators of failing dialysis function, including uremia and poor clearance. Angioplasty was always performed alongside thrombectomy to address stenosis at or adjacent to the site of thrombosis.

Pharmacological thrombolysis with tPA was indicated in the setting of early or small thrombosis of the fistula or graft, where thrombectomy was determined to be too invasive. Thrombolysis was also used alongside balloon angioplasty and stenting in cases where both stenosis and thrombosis occurred at the same location. Flow limitation was assessed with use of digital angiography and intravascular ultrasound (IVUS). Limitation was determined as greater than 50% flow via collateral vessels upon angiography visual inspection, plus concomitant narrowing of the lumen by at least 30% relative to adjacent healthy vasculature seen on IVUS and in some cases, fluoroscopy. Stents were purposefully oversized by 2 mm greater than the diameter of adjacent healthy vessel when feasible, measured by digital angiography measurements or IVUS.

All patients were on some form of anticoagulation at the time of angioplasty or stenting, with the majority on a heparin drip. If heparin was given intra-procedurally, it was dosed to an activated clotting time (ACT) target of 200. Bolus dosing was used in addition to any baseline anticoagulation in increments of 1000 units, based on ACT results, every half-hour. All patients were prescribed an anticoagulant with variable duration of use, typically between 3 and 12 months, according to American Society of Hematology (ASH) guidelines [16].

Chart review was performed for each patient throughout the study period to assess whether they underwent transplant or whether any issues with their dialysis circuit was reported. It was anticipated that not all patients would have imaging after their last intervention if the circuit remained functional.

### Statistics

Descriptive statistics were used to summate baseline patient characteristics and comorbidities. Median, interquartile range, and ranges were used for this unlikely normal distributed small sample size. Additionally, simple ratios and percentages of observations were also performed. In addition, Kaplan–Meier survival analyses were performed for time to reintervention as a full sample and stratified by different phase of reintervention.

## Results

Twelve of seventeen patients were male (70.4%) with a median age at first intervention of 13 years (IQR 10.1–16.9, range 7–18) and median weight in kilograms of 47 (IQR 25.9–63.4, range 22–143). Comorbidities were compiled and measured by prevalence (Table 1). Most patients (59%) had grafts while 41% had fistulas. Dialysis circuit locations were as follows: 47.1% in the left upper extremity, 41.2% in the left lower extremity, and 5.9% each in the right upper or lower extremity.

**Table 1:** Baseline patient characteristics

	Percentage
Male	71%
Hypertension	59%
Anemia	47%
Renal Osteodystrophy	29%
Asthma	18%
Prior transplant	18%
FSGS	24%
RPGN	6%
CAKUT/PUV	29%
Diabetes	12%

**Fig. 1:**
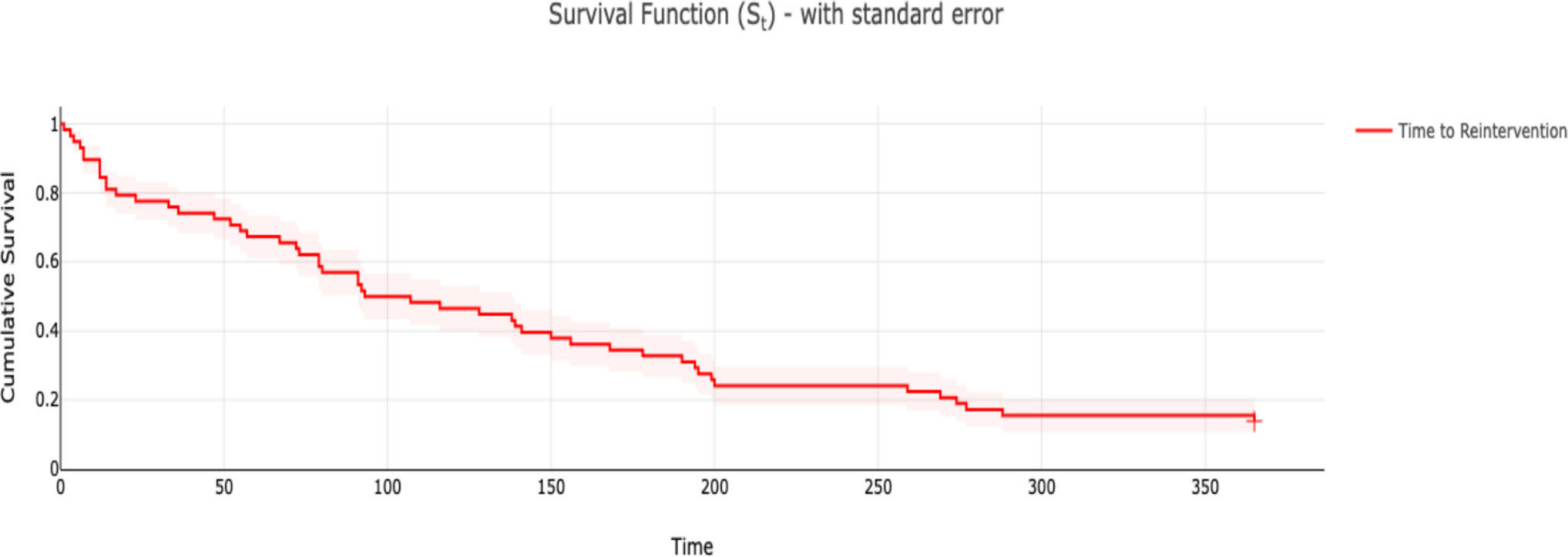
Dialysis circuit reintervention analysis. Kaplan–Meier curve of time to reintervention for all dialysis circuit interventions

**Fig. 2:**
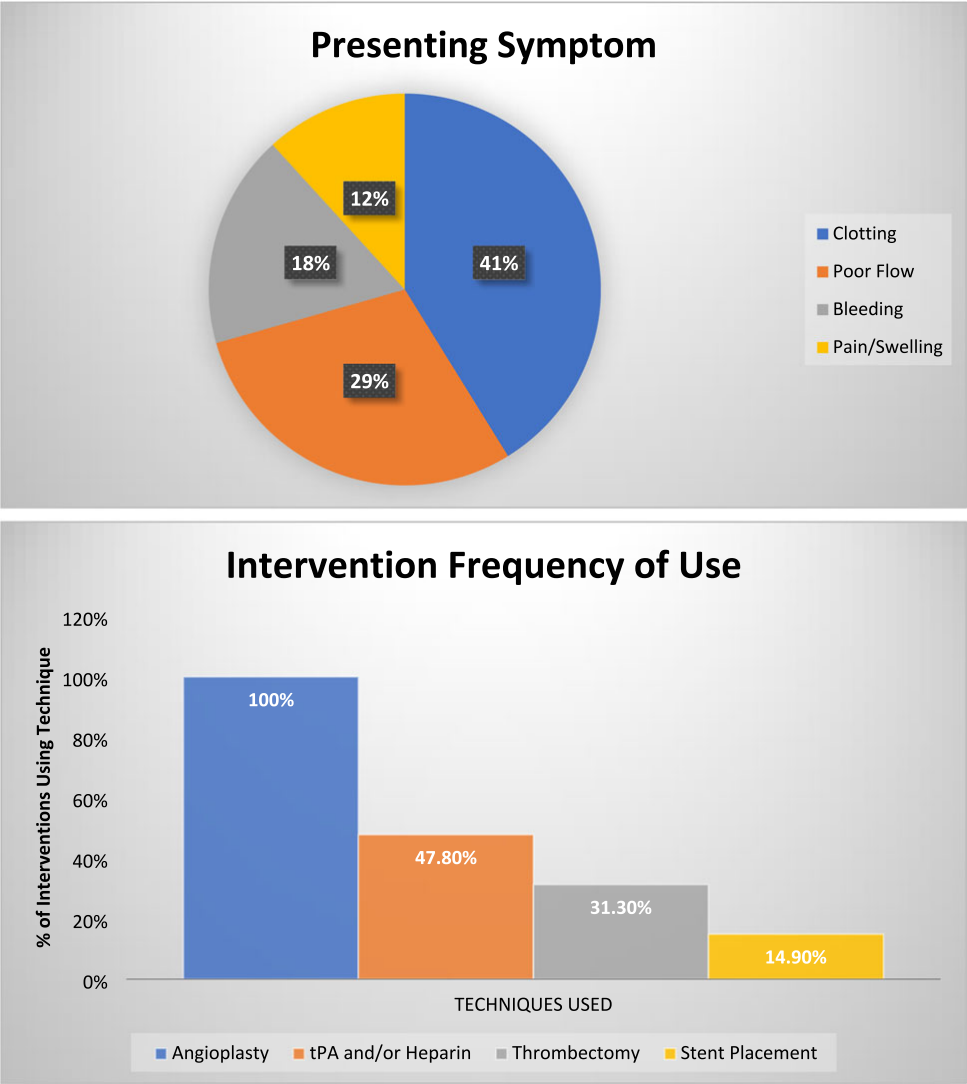
Presenting symptoms. Frequency of presenting complaint for which dialysis circuit intervention was performed and frequency of use for different types of interventions

**Fig. 3:**
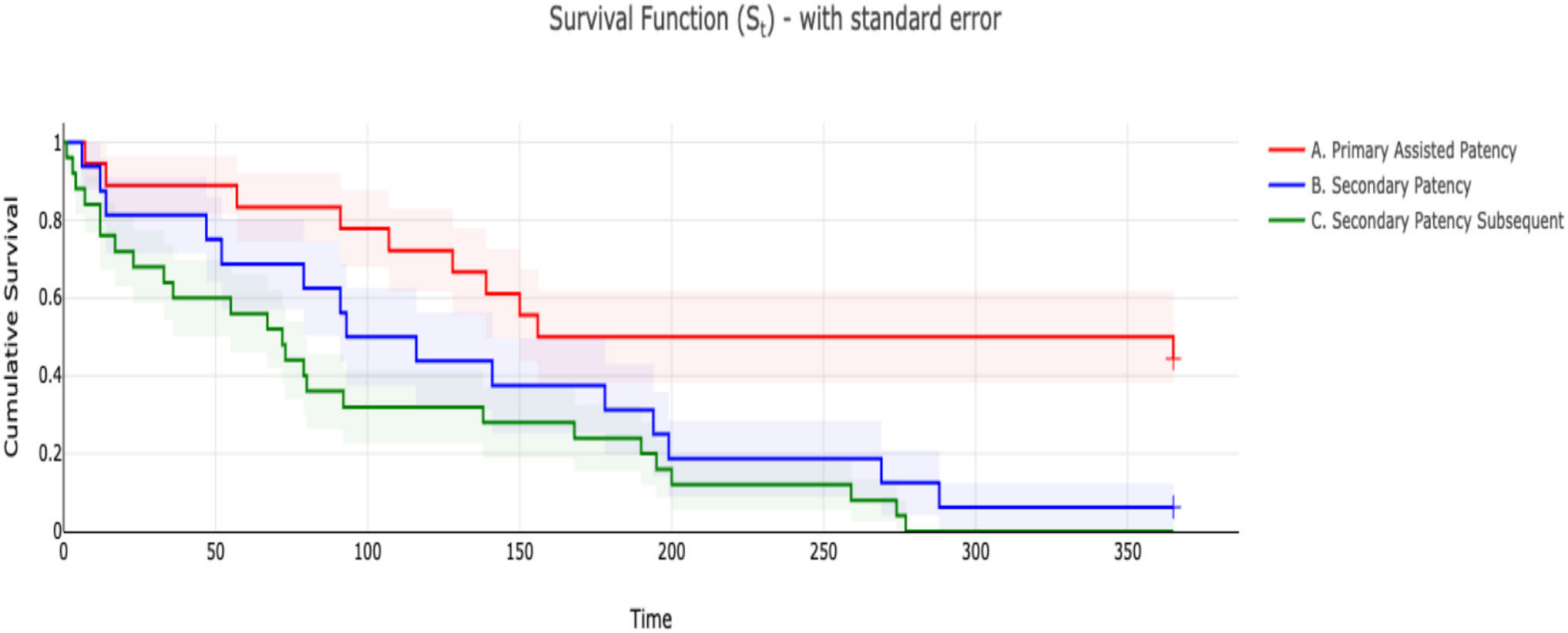
Dialysis circuit reintervention analysis stratified. Kaplan–Meier curves of time to reintervention as divided by primary assisted patency, secondary patency, and subsequent secondary patency

Technical success was 98.5% (66/67), and combined 6-month primary assisted and secondary patency was 41.8% (28/67) across all procedures [Fig. 1]. The one technical failure occurred due to inability to advance the guidewire through the target anatomy (long-segment stenosis in the common femoral vein). This patient had failed renal transplant years prior and, at the time of intervention, had a graft, but was later transitioned to a native fistula due to graft failure. No major or minor complications were observed during or after the procedures. Patients’ initial interventions were categorized by clinical presenting symptom [Fig. 2]. Ten out of seventeen (59%) of patients required at least one reintervention and 7/17 (41%) required at least three [Figs. 1 and 3]. The median number of interventions was 2 (IQR 1–4, range 11), and the median time to first reintervention was 118 days (IQR 65.5–147.25, range 509). For subsequent reinterventions, the median interval was 79 days (IQR 21.5–181, range 287) (Fig. 3). The one patient requiring 12 interventions received angioplasty 12 times, had 2 stents placed, and underwent thrombectomy twice. This patient was 9 years old at the time of initial intervention and carried a very significant burden of comorbidities, including premature birth, posterior urethral valves, bronchopulmonary dysplasia, obstructive sleep apnea, type 1 diabetes, asthma, renal osteodystrophy, and renal transplant rejection. At the time of submission, 76.5% (13/17) of patients have received a renal transplant, one of which occurred within 6 months of the patient’s most recent procedure to preserve access maintenance. The other four patients remain on the waitlist for transplantation, including one pediatric patient and three who have since transitioned to adult units.

A specific breakdown of the combinations of techniques used by procedure is included (Table 2). Kaplan–Meier curves were calculated for overall time to reintervention and were also stratified by primary assisted patency, secondary patency, and subsequent secondary patency [Figs. 1, 3]. Angioplasty balloon diameters ranged from 4 to 10 mm; 96 distinct balloons were utilized across the 63 procedures in which angioplasty was employed—balloons were sized 2 mm greater than the diameter of adjacent healthy vasculature, measured by angiography or IVUS. 35.3% (6/17) of patients received at least one stent, with a total of 12 stents placed across the 10 procedures in which stenting occurred. Stent diameter was 8 mm in 60% of stents, 9 mm in 20%, and 4 mm and 7 mm in 10% each, with lengths ranging from 4 to 6 cm. Patients who underwent stenting had one or fewer subsequent interventions after initial stenting in all but three cases (7/10). Two of the remaining patients had subsequent stenting, of which one had no further reintervention and the second had two additional reinterventions prior to durable patency. The final patient is the aforementioned patient with a total of 11 interventions. For stented patients requiring reintervention, there was a median of 55 days between interventions (IQR 12–138, range 1–199). Dose of tPA ranged from 2 to 4 mg based upon thrombus size, while heparin was dosed to achieve an ACT of 200, with dose ranging from 1000 to 5000 units total for a given procedure.

**Table 2:** Techniques employed for dialysis intervention

Combination of techniques	Number of procedures	Percentage of total procedures
Angioplasty	30	48%
Angioplasty + Thrombectomy + Thrombolysis	14	21%
Angioplasty + Thrombolysis	12	18%
Angioplasty + Thrombectomy + Thrombolysis + Stenting	6	9%
Angioplasty + Thrombolysis + Stenting	3	5%
Angioplasty + Thrombectomy	1	2%
Angioplasty + Stenting	1	2%

## Discussion

The safety and feasibility of percutaneous endovascular techniques in the pediatric population are poorly described in the literature, in part due to the markedly lower prevalence of pediatric ESRD, compounded by the fact that dialysis serves a non-permanent role as a bridge to transplant.

The findings of this study suggest that the techniques discussed may be both safe and effective for maintaining pediatric dialysis accesses. It is notable that no complications were observed during or after any of the 67 interventions. In adults, rates of complication in angioplasty use for dialysis access have been reported to be 3% to 5% [17]. Though no complications were observed in the 67 interventions examined, great care must still be taken as these techniques are used in future management. Some of the most-observed complications of the described endovascular techniques include vascular injury (dissection, perforation, rupture), infection, and bleeding, either at the access site or due to systemic thrombolysis or anticoagulation. Congenital anomalies that contribute to ESRD, such as CAKUT, impact physiology in complex ways. Such conditions may impact the discrepancy between the technical and clinical success rates observed, as well as the gap between clinical success rates in children and adults [18]. A lack of protocol-driven decision-making may impact long-term success in unknown ways. Interventionalists might prioritize minimizing harm over efficacy, favoring lower complication risk at the cost of long-term patency—or, conversely, adopt aggressive techniques that enhance patency but increase risks like endothelial damage or hemorrhage. Additionally, incomplete understanding of pediatric ESRD pathophysiology likely contributes to the relatively low long-term patency compared to adult fistulas.

The high burden of comorbidities in these patients makes it difficult to determine whether clinical failure reflects ineffective technique or underlying disease. Pediatric vessels' growth potential, unlike in adults, must also be considered when interpreting low clinical success rates. Stent use highlights these challenges, as ongoing growth poses risks of migration and neointimal hyperplasia leading to in-stent stenosis. Angioplasty emerges as a primary option, as it was the markedly the most-employed technique. Studies have shown that in the adult population, use of angioplasty alone for dialysis access maintenance resulted in clinical success rates of 42–63% for fistulas and 27–61% for grafts [17]. The observed rate of combined 6-month primary assisted and secondary patency in this study was 41.8% across all interventions, and it would not be possible to extricate the impact on 6-month patency of angioplasty alone. However, as angioplasty was employed in every intervention, adult patency rates provide an important datapoint for comparison.

Stent placement served as a supplement in the relatively few cases where angioplasty alone was insufficient to resolve stenosis. Stents were also used to provide structural support to vasculature refractory to angioplasty to minimize the need for future reintervention. Stents were also indicated for symptomatic relief of pain and swelling in such areas unresponsive to angioplasty alone. All six patients receiving stents had undergone multiple previous procedures involving angioplasty.

Risk of vascular trauma and subsequent acute or chronic thrombosis must be acknowledged when selecting a location for stent placement and considering perioperative and long-term pharmacological management. Introduction of any stent, particularly a bare metal stent over a drug-eluting stent, can predispose to recurrent thrombus [19]. Patency rates may have been impacted by development of thrombus at stent sites, as suggested by the patients who received one or more stents and later received heparin and/or tPA.

Use of pharmacological thrombolytics and rotational thrombectomy devices was complimentary and was shown to be safe and feasible. The combined use of alteplase and percutaneous rotational thrombectomy devices was effective in clot dissolution in the setting of large or resistant thrombus. Possible adverse events especially relevant to these techniques are embolization of thrombotic debris once dislodged [20]. Ways to mitigate risk of these events occurring include thorough maceration of thrombus to the point that any debris is of insignificant size and intraprocedural placement and removal of an inferior vena cava filter.

Risks must still be addressed, even though no complications were observed. While percutaneous access is minimally invasive, wire-guided angioplasty, stenting, and thrombectomy can disrupt stenotic plaques or thrombi, leading to local vascular trauma. Pharmacologic thrombolytics also carry bleeding risks at the treatment site or systemically. These risks were minimized through careful anticoagulant and antiplatelet selection, conservative dosing, and individualized access site choice. Extensive operator experience in adults lent itself toward greater care in pediatric interventions. Additionally, pediatric vessels may be more resilient than adult vessels, which often suffer from chronic inflammation due to smoking, diabetes, or ESRD. Several limitations must be acknowledged. First, the overall sample size is low, due at least in part to the rarity of pediatric patients requiring a fistula or graft. However, the study was intended to explore safety and feasibility and for those aims the sample size does seem acceptable. The relative effectiveness of each individual technique is also difficult to discern given the variability of use in isolation or conjunction with other techniques. Finally, the use of intention to treat, necessary due to the lack of follow-up common in this patient population, like their adult correlates, limits the full capture of long-term outcomes.

## Conclusion

This study suggests that endovascular interventions for pediatric dialysis access maintenance and salvage—including angioplasty, stenting, thrombectomy, and thrombolysis—are safe, effective, and yield high technical success. However, while consistent with prior observations, the 6-month primary assisted and secondary patency rate in pediatric patients is slightly lower than in adults, averaging 41.8% compared to approximately 60% reported in adult literature.

## Conflict of interest

The authors of this paper have no conflicts of interest to declare.

## Ethics approval

This retrospective chart review study involving human participants was in accordance with the ethical standards of the institutional and national research committee and with the 1964 Helsinki Declaration and its later amendments or comparable ethical standards. For this type of study, formal consent is not required. For this type of study, consent for publication is not required. The Human Investigation Committee (IRB) of the University of Alabama at Birmingham approved this study.
